# Genomewide Gene-by-Sex Interaction Scans Identify *ADGRV1* for Sex Differences in Opioid Dependent African Americans

**DOI:** 10.1038/s41598-019-53560-0

**Published:** 2019-12-02

**Authors:** Bao-Zhu Yang, Hang Zhou, Zhongshan Cheng, Henry R. Kranzler, Joel Gelernter

**Affiliations:** 1 Yale University School of Medicine, Department of Psychiatry, New Haven, CT USA; 2 VA Connecticut Healthcare System, Department of Psychiatry, West Haven, CT USA; 3 University of Pennsylvania Perelman School of Medicine, Department of Psychiatry, Philadelphia, PA USA; 4 VISN 4 MIRECC, Crescenz Philadelphia VAMC, Philadelphia, PA USA; 5 Yale University School of Medicine, Departments of Genetics and Neuroscience, New Haven, CT USA

**Keywords:** Gene regulatory networks, Microarrays, Behavioural genetics

## Abstract

Sex differences in opioid dependence (OD) are genetically influenced. We conducted genomewide gene-by-sex interaction scans for the DSM-IV diagnosis of OD in 8,387 African-American (AA) or European-American subjects (43.6% women; 4,715 OD subjects). Among AAs, 9 SNPs were genome-wide significant at *ADGRV1* (*adhesion G-protein-coupled receptor V1*, lead-SNP rs2366929*(C/T), *p* = 1.5 × 10^−9^) for sex-different risk of OD, with the rs2366929*C-allele increasing OD risk only for men. The top co-expressions in brain were between *ADGRV1* and *GRIK*2 in substantia nigra and medullary inferior olivary nucleus, and between *ADGRV1* and *EFHC*2 in frontal cortex and putamen. Significant sex-differential *ADGRV1* expression from GTEx was detected in breast (Bonferroni-corrected-*p* < 0.002) and in heart (*p* < 0.0125), with nominal significance identified in brain, thyroid, lung, and stomach (*p* < 0.05). *ADGRV1* co-ex*p*ression and disease-enrichment analysis identifying the top 10 diseases showed strikingly sexually dimorphic risks. The enrichment and transcriptome analyses provided convergent support that *ADGRV1* exerts a sex-different effect on OD risk. This is the first study to identify genetic variants contributing to sex differences in OD. It shows that *ADGRV1* contributes to OD risk only in AA men, a finding that warrants further study.

## Introduction

Sex differences in opioid dependence (OD) have a strong biological basis^1^ with a genetic component^2,3^. Heritability estimates of heroin use are around 0.5^2^. Although there is no report for sex-specific heritability estimates of OD specifically, heritability estimates of drug dependence are higher in men than women^4^. In animal models, genetic effects related to OD have differed by sex^5–7^. For example, a mouse model of the human *OPRM1* (A118G) polymorphism found genotype-by-sex-specific reductions in the rewarding properties of morphine^5^. This variant does not, however, appear to affect OD risk in humans^8^. To our knowledge, the only systematic genome-wide search for the genes responsible for sex differences in human OD was a linkage study^3^.

Systematic search for the OD genetic risk variants (regardless of sex differences) using the genome-wide association study (GWAS) design has been reported. We published the first GWAS with genomewide significant findings^9^. The most compelling results in that study were the identification of genes involved in potassium signaling pathways (i.e., *KCNC1* (*Potassium Voltage-Gated Channel Subfamily C Member 1*) and *KCNG2* (*Potassium Voltage-Gated Channel Modifier Subfamily G Member 2*)) in the African American (AA) population, and genes involved in calcium signaling and long-term potentiation. Another GWAS was conducted in an Australian cohort, in which genetic data from opioid-dependent daily injectors were compared with that from opioid misusers who never progressed to daily injection, and identified several genomewide significant variants in *CNIH3* (*Cornichon Family AMPA Receptor Auxiliary Protein 3*)^10^. In a recent study, we found that a variant on chromosome 15, rs12442183, near *RGMA* (*Repulsive Guidance Molecule A*), was genome-wide significantly associated with OD in the European American (EA) population^11^. *RGMA* encodes a central nervous system axon guidance protein called repulsive guidance molecule A. Risk allele rs12442183*T was related with higher expression of a specific *RGMA* transcript variant in frontal cortex. After chronic morphine injection, the homologous mouse gene, *Rgma*, was upregulated in the striatum of C57BL/6 J mice^11^.

We aimed to identify genetic variants exerting a sex difference in susceptibility to OD in a GWAS framework using the cohort of substance use disorder we have collected; characterize and annotate the identified genetic variants using publicly available databases of co-expressed genes and enrichment analysis; and use transcriptome analysis to identify biological mechanisms consistent with sex-specific effects on OD risk for the variants identified.

## Materials and Methods

### Subjects and the semi-structured assessment

This study analyzed data obtained from African-American (AA) and European-American (EA) participants in the Yale-Penn genetics of substance dependence cohort (total N = 8,387). The 4,944 AA and 3,443 EA samples were recruited from 2000 to 2013, as previously described^9,12^. These subjects represent two phases of collection: Yale-Penn-1 (N = 4,970) and Yale-Penn-2 (N = 3,417), which differed in their period of recruitment and the genotyping platforms used. The demographic characteristics of the study cohort are presented in Table 1. We assessed all subjects using the Semi-Structured Assessment for Drug Dependence and Alcoholism (SSADDA)^13^ and obtained certificates of confidentiality for all subjects from the National Institute on Drug Abuse (NIDA) and the National Institute on Alcohol Abuse and Alcoholism (NIAAA). The study protocol was approved by the Yale Institutional Review Board and the study was performed in accordance with the relevant guidelines and regulations. All subjects provided written informed consent

**Table 1:** Demographic characteristics of the study samples.

Characteristic	African American			European American			Total
	Yale-Penn 1	Yale-Penn 2	Subtotal	Yale-Penn 1	Yale-Penn 2	Subtotal	
N Male, N Female, N Female %	3,227 1,707 1,520 47.10%	1,717 1,001 716 41.70%	4,944 2,709 2,235 45.20%	1,743 1,011 732 42%	1,700 1,013 687 40.40%	3,443 2,024 1,419 41.20%	8,387 4,730 3,657 43.60%
Age (SD)	41.1 (9.0)	40.8 (10.9)	41.0 (9.7)	38.0 (10.9)	39.4 (13.0)	38.7 (12.0)	40.1 (10.7)
OD affected, N Male, N Female, N Female %	1,684 994 690 41%	919 643 276 30%	2,603 1,637 966 37.10%	1,086 682 404 37.20%	1,026 683 343 33.40%	2,112 1,364 748 35.40%	4,715 2,999 1,716 36.40%
OD unaffected, N Male, N Female, N Female %	1,543 713 830 53.80%	798 358 440 55.10%	2,341 1,070 1,271 54.30%	657 329 328 49.90%	674 330 344 51%	1,331 659 672 50.50%	3,672 1,730 1,942 52.90%

### Genotype quality control, population stratification, and imputation

We genotyped the Yale-Penn-1 sample with the Illumina HumanOmni1-Quad array of approximately 988,000 single nucleotide polymorphisms (SNPs), and the Yale-Penn-2 sample with the Illumina HumanCore Exome array of approximately 266,000 exonic SNPs and 240,000 tagging SNPs. We excluded SNPs with a genotype call rate <98% or minor allele frequency (MAF) <1%.

We used the following measures to differentiate AA or EA subjects and control for population stratification. First, we conducted principal component (PC) analysis^14^ for the SNPs common among the genetic data for the Yale-Penn-1, Yale-Penn-2, and the 1000 Genomes phase 3 reference panel, which contains African, admixed American, European, East Asian and South Asian populations^15^. We then trimmed SNPs in LD (*r*^2^ > 0.2) using PLINK^16^. Using the remaining SNPs, we clustered the Yale-Penn subjects into different groups compared to the reference populations by the first three PCs in Euclidean space. Subjects were removed from the subsequent analyses if they were not clustered with African or European populations. Finally, we conducted a second PC analysis within each group to remove outliers greater than three standard deviations from the mean Euclidean distances. The resultant first 10 PCs were covariates in all subsequent association analyses to adjust for residual population stratification.

We imputed GWAS data to the 1000 Genomes phase 3 reference panel^15^, using Minimac3 implemented in the Michigan Imputation Server^17^. Post-imputation SNP exclusion metrics included: Hardy-Weinberg equilibrium *p < *10^−6^, imputation accuracy < 0.8, or MAF < 5%. The final SNP counts for the subsequent association analyses were: Yale-Penn-1 sample, 8,775,706 SNPs in AAs and 6,417,418 in EAs; Yale-Penn-2 sample, 6,702,161 SNPs in AAs and 5,205,763 in EAs.

### Genomewide gene-by-sex interaction scan for sex differences in OD

We performed genetic association tests for the DSM-IV diagnosis OD using a linear mixed model implemented in the program GEMMA^18^. A standard linear mixed-effect model was chosen to control for the relatedness among participants as our cohort contained a mixture of individuals ascertained using both an unrelated case-control design and a family design (11.9% of AAs and 7.9% of EAs came from the family design). We chose the standard linear mixed effect model implemented in GEMMA, where the effect estimates asymptotically converge to estimates of logistic regression for a binary outcome (such as OD affected versus OD unaffected here) when the sample size is large. We tested the gene-by-sex interaction effect and included the main effects of sex and SNP in addition to controlling for age, four other substance dependence diagnoses (alcohol, cocaine, nicotine, cannabis) and the first 10 PCs, to examine sex differences in genetic risk for OD. This model was examined separately for each of four datasets, i.e., both AA and EA subjects in the Yale-Penn-1 and Yale-Penn-2 samples. We then meta-analyzed the interactive effects of SNP-by-sex from Yale-Penn-1 and Yale-Penn-2 within each population using the inverse variance method that converted all the effects into a signed Z-score and tested for association using the Z-test, which was implemented in the program METAL^19^.

### Co-expression, sex differences in gene expression, disease enrichment, and functional annotation

For the enrichment analysis, we first queried the target gene, i.e., the gene harboring the genomewide significant variants detected, to identify co-expressed genes using COXPRESdb^20^, a coexpression database of DNA-microarray and RNAseq-based expression data. After obtaining the top 100 co-expressed genes of the targeted gene (or genes) identified from the association analyses, we assessed functional enrichment using the WEB-based GEne SeT AnaLysis Toolkit (WebGestalt)^21^, a functional enrichment analysis web tool (see supplementary material for the setting of parameters). WebGestalt 2017 supports 12 organisms and 150,937 functional categories from public databases and computational analyses^21^. The enrichment analysis method ‘Disease Association Analysis’ of WebGestalt was used to test for enrichment of disease-associated genes among the top co-expressed genes with the targeted gene.

We studied sex differences in the expression of the targeted gene, i.e., *ADGRV1*, to investigate the functional roles of the top variants and the disease-enriched genes to provide insight into the molecular mechanism of sex differences in OD. To do this, we used the Genotype-Tissue Expression (GTEx) data and the tissue-specific gene expression database, the Brain eQTL Almanac, Braineac^22^. The RNA-Seq data of GTEx Analysis V7 (phs000424.v7.p2), included in the file, ‘GTEx_Analysis_2016-01-15_v7_RNASeQCv1.1.8_gene_tpm.gct.gz’, were downloaded from the GTEx Portal datasets^23^, and information about tissues and sex was retrieved from the files, ‘GTEx_v7_Annotations_SampleAttributesDS.txt’ and ‘GTEx_v7_Annotations_SubjectPhenotypesDS.txt’, respectively. We downloaded the gene expression (RNA-seq) data and the related phenotypes and analyzed the data using the general linear model, implemented in the SAS procedure “ proc glm,” where sex was modeled as the main effect in association with the levels of gene expression.

## Results

### Genomewide gene-by-sex interaction scan

**Figure 1:**
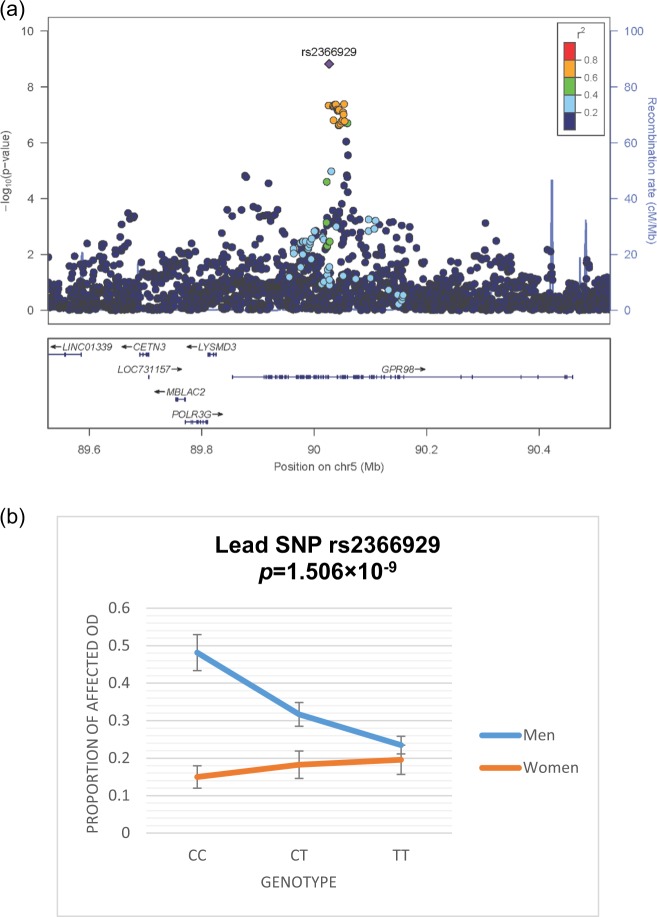
Genomewide gene-by-sex interaction scans on opioid dependence (OD). (**a**) Regional association plot at the *ADGRV1* (aka. *GPR98*) locus for the meta-analysis of the two phases of the African American (AA) sample. (**b**) Proportions of the OD affected for the AA sample for each genotype for the lead SNP rs2366929.

We summarized the result for each dataset and the meta-analysis within each population in the Manhattan plots (Supplementary Figure S1). The QQ plots show no systematic bias (Figure S1). A peak with the lead SNP rs2366929 and another eight SNPs in strong LD (*r*^*2*^ > 0.9) with the lead SNP (*r*^*2*^ > 0.8) reached genomewide significance (all *p’s* < 5 × 10^−8^) in the gene-by-sex interaction scans for OD in the meta-analyzed AA samples (Fig. 1a). For lead SNP rs2366929, each subsample also showed dose-response effects of nominal significance with *p* = 2.19 × 10^−5^ and *p* = 1.23 × 10^−5^ for Yale-Penn 1 and Yale-Penn 2, respectively. This peak of genomewide significance is located at *ADGRV1* (*Adhesion G protein-coupled receptor V1*) on chromosome 5q14.3. The gene-by-sex interaction effects exhibit a gene dose-response relationship for the OD risk in AA men but not women (Fig. 1b). That is, the proportions of OD-affected to the total subjects (OD affected plus controls), within each of the three genotypes consistently show a gene dose-response trend relationship in men but not women (for lead SNP rs2366929*(C/T), chi-square test for trend in proportions, *p* = 1.1 × 10^−14^ in men and *p* = 0.15 in women). Figure 1b displays the increasing trend of the OD-affected proportions for men (CC > CT > TT) compared with women for the lead SNP rs2366929. This increasing trend in men versus women was observed for all nine SNPs identified in the association analyses (Supplementary Table S1 shows the trend tests for all nine SNPs). The MAF for the lead SNP increased 10.5% for the OD-affected versus the OD-unaffected men (0.353 vs. 0.248; comparison of two proportions, *p < *0.0001); however, this increase was not present in women (0.241 vs. 0.265; *p* = 0.15). Table 2 displays the characteristics of the nine genome-wide significant SNPs.

**Table 2:** Genomewide significant SNPs located at the *ADGRV1* locus with differential effects on susceptibility to opioid dependence between male and female for the African American sample.

SNP	SNP rsID	Position on Chr5/GRCh37	LD (*r*^2^)	Distance from the lead SNP (bp)	Alleles	Male		Female		Beta*	Standard error*	Meta *p*-value
						MAF OD Affected N = 1637	MAF OD Unaffected N = 1070	MAF OD Affected N = 966	MAF OD Unaffected N = 1271			
1	rs2030272	90025842	0.75	−792	C/G	0.31	0.213	0.209	0.222	0.10	0.019	4.55e−08
2	rs2366929	90026634		0	C/T	0.353	0.248	0.241	0.265	0.11	0.018	1.51e−09
3	rs2222243	90033697	0.75	7063	A/C	0.31	0.213	0.209	0.223	0.10	0.019	4.96e−08
4	rs2443067	90034302	0.75	7668	A/G	0.31	0.213	0.209	0.223	0.10	0.019	4.96e−08
5	rs2443066	90034784	0.73	8150	A/G	0.31	0.213	0.209	0.223	0.10	0.019	4.49e−08
6	rs2460186	90036980	0.75	10346	A/G	0.311	0.213	0.209	0.224	0.10	0.019	4.21e−08
7	rs2460187	90038450	0.72	11816	G/T	0.311	0.213	0.209	0.224	0.10	0.019	4.21e−08
8	rs2443065	90053056	0.70	26422	A/G	0.313	0.216	0.211	0.227	0.10	0.019	4.04e−08
9	rs2443064	90053280	0.70	26646	T/C	0.313	0.216	0.211	0.227	0.10	0.019	4.04e−08

Note: MAF, Minor Allele Frequency; OD, Opioid Dependence (DSM-IV diagnosis); SNP, Single Nucleotide Polymorphism. *Beta (Standard error) was derived for the SNP-by-sex interaction effect from the METAL which pulled together the results from the GEMMA software program implementing the linear mixed effect model for Yale-Penn-1 and Yale-Penn-2 (as described in the method section).

We identified no genomewide significant signals in the EA sample (Supplementary Figure S2) and the trans-ethnic meta-analysis of the AA and EA samples (Supplementary Figure S3).

### Sex difference in *ADGRV1* gene expression

We used GTEx data to identify tissues with differential *ADGRV1* gene expression by sex for further annotating *ADGRV1*. We detected significant sex-differential *ADGRV1* gene expression in breast (Bonferroni correction *p* < 0.002) and in heart (*p* < 0.0125) based on GTEx transcriptome data (dbGaP Accession phs000424.v7.p2) (Table 3). The *ADGRV1* expression in heart supports a previous study of sexually dimorphic *ADGRV1* expression in patients with non-ischemic human heart failure (Supplementary Table S2), in which *ADGRV1* expression was lower in men than women, the same effect direction to what we found in the GTEx transcriptome. We identified four additional tissues with sex-differential *ADGRV1* gene expression that were nominally significant, including brain (*p* = 0.018), thyroid (*p* = 0.021), lung (*p* = 0.038), and stomach (*p* = 0.045) (Table 3).

**Table 3:** *ADGRV1* gene expression: Tests of sexually dimorphic expression using Genotype-Tissue Expression (GTEx) project data.

Tissue	Average Gene Expression^a^ (N)		df	F Value	Pr > F
	Male	Female			
Adipose Tissue	0.031 (526)	0.025 (271)	(1, 795)	1.46	0.227
Adrenal Gland	62.92 (108)	53.50 (82)	(1, 188)	2.67	0.104
Bladder	1.31 (6)	0.76 (5)	(1, 9)	0.66	0.439
Blood	0.089 (349)	0.070 (188)	(1, 535)	0.36	0.546
Blood Vessel	0.042 (593)	0.036 (320)	(1, 911)	0.13	0.722
*Brain*	***6.06 (1162)***	***6.81 (509)***	***(1, 1669)***	***5.6***	0.0181*
*Breast*	***0.65 (175)***	***1.33 (115)***	***(1, 288)***	***35.27***	<0.0001**
Colon	0.10 (306)	0.11 (201)	(1, 505)	1.87	0.173
Esophagus	0.12 (647)	0.12 (374)	(1, 1019)	0.21	0.644
*Heart*	***0.018 (402)***	***0.025 (198)***	***(1, 598)***	***6.28***	0.0125*
Kidney	0.50 (36)	0.37 (9)	(1, 43)	0.38	0.542
Liver	0.59 (120)	0.58 (55)	(1, 173)	0	0.962
*Lung*	***1.42 (286)***	***1.61 (141)***	***(1, 425)***	***4.33***	0.038*
Muscle	0.043 (371)	0.039 (193)	(1, 562)	0.17	0.684
Nerve	0.10 (274)	0.10 (140)	(1, 412)	0.11	0.738
Pancreas	0.43 (150)	0.45 (98)	(1, 246)	0.75	0.386
Pituitary	9.96 (129)	9.95 (54)	(1, 181)	0	0.995
Salivary Gland	0.62 (71)	0.53 (26)	(1, 95)	1.64	0.203
Skin	0.17 (790)	0.17 (413)	(1, 1201)	0.01	0.909
Small Intestine	0.070 (86)	0.059 (51)	(1, 135)	1.24	0.268
Spleen	0.03 (98)	0.02 (64)	(1, 160)	0.61	0.436
*Stomach*	***0.16 (160)***	***0.20 (102)***	***(1, 260)***	***4.08***	0.0445*
*Thyroid*	***6.91 (293)***	***8.42 (153)***	***(1, 444)***	***5.36***	0.0211*

Note: ^a^The gene expression unit is transcripts per million (TPM); df, degrees of freedom; N, sample size; Pr, probability; **p < 0.002 (Bonferroni correction for 23 tests); *p < 0.05.

### *ADGRV1* co-expression and disease enrichment analysis

The top 100 genes co-expressed with *ADGRV1* (Supplementary Table S3) identified by COEXPRESdb were subject to disease enrichment analysis. We found that *ADGRV1* and 13 *ADGRV1* co-expressed genes were significantly enriched within 10 diseases (all raw *p’s* < 0.01 and adjusted *p’s* < 0.05, Supplementary Table S4). The 13 genes are *ABCC12*, *CFTR*, *CTAGE1*, *DAOA-AS1*, *EFHC2*, *FHL5*, *GRIK2*, *NAV3*, *SPO11*, *SYCP2*, *TAAR9*, *TPH2*, and *ZNF157*. Among the 10 diseases, two are male-specific (male infertility and non-syndromic oligospermia), four affect males more than females (X-linked mental retardation, generalized epilepsy, Sezary syndrome, cutaneous T-cell lymphoma), three affect females more than males (chronic fatigue syndrome, panic disorder, and pseudoxanthoma elasticum), and one displays distinct disease characteristics between males and females (personality disorders).

**Figure 2:**
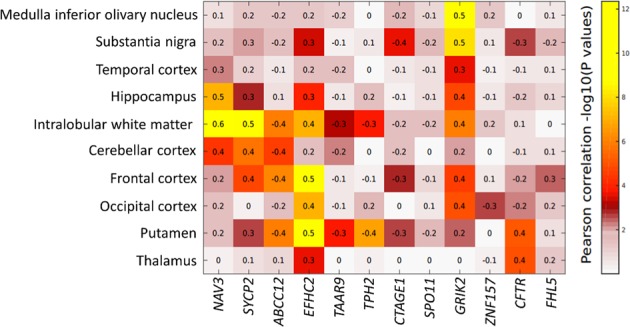
The mRNA expression of *ADGRV1* was associated with 12 co-expressed genes across 10 human brain tissues in the Braineac database (Ramasamy *et al*.^22^). Pairwise Pearson correlation coefficients (*r*) for *ADGRV1* and the 12 genes are labeled in each square of the heatmap. The color bar indicates the extent of significance, $-{\log }10(p-{values})$, for the correlation coefficients.

We also correlated the expression of *ADGRV1* with the expression of the 12 co-expressed disease risk genes (*DAOA-AS1* expression was not available) across the 10 human brain tissues in the Braineac database. The correlation pattern of gene expression across the 10 brain tissues is displayed in Fig. 2. Robust correlations of gene expression between *ADGRV1* and two genes, *GRIK2* (Glutamate ionotropic receptor kainate type subunit 2) and *EFHC2* (EF-hand domain containing 2), are ubiquitous across various human brain tissues. The highest correlations between the gene expressions of *ADGRV1* and *GRIK2* are in the substantia nigra and medulla inferior olivary nucleus and between *ADGRV1* and *EFHC2* in the frontal cortex and putamen (Fig. 2).

## Discussion

To our knowledge, this is the first systematic search for genetic variants contributing to sex differences in OD risk using GWAS. We identified a male-specific effect of *ADGRV1* on risk of OD in the AA sample using genome-wide gene-by-sex interaction scans. Co-expressed genes, the enrichment analysis, and the transcriptome analysis provided mechanistic support for the finding that *ADGRV1* exerts a sex-specific effect on OD risk.

*ADGRV1* spans approximately 605.5 kb on chromosome 5q14.3 and encodes a member of the G-protein coupled receptor superfamily. In addition to the N- and C-terminal domains of ADGRV1, the encoded protein contains 7 putative Na( + )/Ca(2 + ) exchangers (defining the cation binding domain) and 21 N-linked glycosylation sites^24^. Its extracellular repeat domains bind Ca(2 + ) and are involved in signal transduction^24^. Members of the ADGR family play key roles in regulating cortical patterning, dendrite and synapse formation, and myelination^25^. Using GTEx transcriptome data and the Human Protein Atlas^26^, we found that *ADGRV1* is also highly expressed in three endocrine glands (adrenal gland, thyroid, pituitary), lung, and multiple brain regions (Supplementary Figure S4). Opioid use disrupts hypothalamic-pituitary-adrenal (HPA) dynamics at the level of the pituitary or adrenal^27^. Chronic opioid use can cause pervasive endocrine dysfunction, which leads to hypogonadism, infertility, fatigue, anxiety, depression, menstrual irregularities, and so on^28,29^. Many of these disorders are among what we have identified for this study in the top ten diseases significantly enriched by the *ADGRV1* co-expressed genes, such as fatigue, infertility, anxiety or panic disorder (Supplemental Table S4).

In the co-expressed genes and enrichment analyses, all of the top 10 diseases identified demonstrate sexually dimorphic risks or manifestations. We identified *ADGRV1* as a male-specific risk factor in OD. The top 10 diseases also include two that are male-specific (i.e., male infertility and non-syndromic oligospermia) or four that predominantly affect males more than females (i.e., X-linked mental retardation, generalized epilepsy, Sezary syndrome, or cutaneous T-cell lymphoma). On the other hand, three of the 10 diseases affect more females than males (chronic fatigue syndrome, panic disorder, and pseudoxanthoma elasticum)^30,31^. Collectively, the top 10 diseases for *ADGRV1* co-expressed genes are strikingly sexually dimorphic.

In disease-association studies, *ADGRV1* has been well examined. Mutations in *ADGRV1* are associated with familial febrile seizures^32^, and also contribute to focal and generalized epilepsy^33,34^, and epilepsy with myoclonic seizures^35^. Besides, *ADGRV1* is associated with cardiac conduction disorder^33^ and *ADGRV1* variants segregated in families with epilepsy co-occurring sudden death (due to cardiac conduction disorder), showing shared *ADGRV1* risk variants between epilepsy and cardiac conduction disorder^33^. *ADGRV1* expression in the heart has been reported for patients with non-ischemic heart failure (Supplementary Table S2), where expression was lower in men than women^36^. This sex difference in *ADGRV1* expression in the heart was replicated in the GTEx transcriptome data (Table 3), although disease status was not controlled in this analysis because of the disease heterogeneity of GTEx donors. *ADGRV1* has also been implicated in adverse metabolic effects of antipsychotic drugs^37^. Taken together, our novel discovery of *ADGRV1* for contributing to OD risk in men add into this scientific literature on the disease-gene association of the strong candidate gene *ADGRV1*.

In the GTEx data, individual-level data do not include the diagnosis of OD. Thus our investigation into the association between sex differences in *ADGRV1* expression and those in OD is not possible in that dataset. However, *ADGRV1* is highly expressed in endocrine tissues (Supplementary Figure S4); opioid use could act on the tissues in which *ADGRV1* is highly expressed (i.e., endocrine and brain), disrupting the normal G-protein coupled receptor signaling and hormone production and causing pathogenic cellular processes. The differences in *ADGRV1* expression could affect stress responses or produce hormonal or behavioral effects that differ by sex in opioid users.

Regarding the co-expression patterns for the enriched genes expressed in brain, the identified brain regions show supportive evidence for neuropathology. The robust correlations in gene expression between *ADGRV1* and *GRIK2* and *EFHC2* are ubiquitous in human brain. The top correlations in gene expressions between *ADGRV1* and *GRIK2* are in the substantia nigra and medulla inferior olivary nucleus (Fig. 2). The substantia nigra is enriched in dopaminergic neurons and plays an important role in reward^38^, while the medulla inferior olivary nucleus is implicated in motor learning and control^39^. Another set of top correlations in the expression are between *ADGRV1* and *EFHC2* in frontal cortex and putamen (Fig. 2). The frontal cortex contains most of the dopamine-sensitive neurons in the cerebrum and is associated with reward, attention, short-term memory, planning, and motivation^40^. The putamen is involved in reinforcement learning and various movements^41^. These results support a role for *ADGRV1* in networks of co-expressed genes that regulate the neural activities involved in addiction-related functions, including reward, memory, and learning.

As for the other co-expressed genes involved in the top correlated brain regions, *TPH2* encodes tryptophan hydroxylase 2, which catalyzes the rate-limiting step in the synthesis of serotonin. Chronic morphine and cocaine exert common effects on tyrosine hydroxylase in dopaminergic brain reward regions^42^. Mutations in *TPH2* were associated with quality of life of patients in methadone maintenance treatment for heroin use disorder^43^, responses to cocaine treatment^44^, and a spectrum of psychiatric disorders (cf. a review and meta-analysis^45^). *GRIK2* encodes a member of the kainate family of glutamate receptor subunits. In a mouse model, *Grik2* mRNA levels were decreased after prolonged morphine treatment^46^. These results again support a role for *ADGRV1* in networks of co-expressed genes that regulate the neural activities involved in opioid or drug addiction.

The variants at *ADGRV1* reaching genomewide significance for a sex-difference in OD risk were identified in AAs, but not EAs. The minor allele “C” of lead SNP rs2366929 in the International Genome Sample Resource (IGSR)^15^ has MAF = 0.47 for Europeans, and MAF = 0.22 for Africans, consistent with the MAFs (0.248 male; 0.265 female) we observed in our admixed AA population (Table 2). Local or population-specific variation is important in mapping disease risk^47,48^. Although the current information is not enough to determine why the genetic effects in *ADGRV1* were not seen in EAs, there are several plausible explanations. For example, effect sizes might be smaller in EA ancestries such that larger sample size may be necessary for discovery. Further studies with larger sample sizes and more power are warranted to investigate sex-differences in OD risk.

The GEMMA model we used for the current study includes 17 main effects (i.e., age, sex, SNP, and four other substance dependence on alcohol, cocaine, marijuana, and nicotine, and ten PCs) and one two-way interaction (i.e., SNP-by-sex). In theory, a saturated model that incorporates all of the two-way interaction effects would be ideal but would involve a total of 153 terms in the model, comprised of the 17 main effects and 136 pairwise interaction effects. We chose not to apply the saturated model because in previous simulations (unpublished data) we obtained effect estimates for the targeted interactions that were generally close to those estimated by the saturated model. However, the power was greatly reduced by the inclusion of a large number of interaction terms. We opted to use the more limited model.

The strengths of the present study include a moderately large cohort of substance dependence subjects who were carefully phenotyped and whose data were rigorously analyzed. However, the study findings are limited by the absence of a suitable cohort of AAs with OD in which to replicate the findings after we made extensive efforts to locate an existing dataset sufficiently powered for replication. Despite this limitation, we believe that the findings from this first study of sex- differences in genetic risk for OD can aid in understanding the underlying differences and facilitate better sex-specific prevention and treatment efforts for OD.

In summary, we identified *ADGRV1* as a risk locus contributing to increased risk of OD in AA men by examining genetic variants systematically on a genome-wide scale. Functional annotation of this finding corroborated a substantial role for *ADGRV1* in increasing OD risk, especially the potential pathogenic effects of variation in *AGDRV1* on cardio-cerebral mechanisms, which could contribute to the risk of fatal opioid overdose or respiratory depression that has been observed following high-dose opioid exposure. Further study of this finding is warranted.

## Supplementary information

Supplementary Information

## Data Availability

The datasets analyzed for the current study are not all publicly available. The Yale-Penn-1 subsample can be requested from the NCBI dbGaP repository: A Genome-Wide Association Study of Heroin Dependence (dbGaP Study Accession: phs000277.v1.p1). The Yale-Penn-2 subsample has not deposited to the dbGaP, but will be available from the corresponding author on reasonable request.
