# Supplementary material for: Genomewide Gene-by-Sex Interaction Scans Identify ADGRV1 for Sex Differences in Opioid Dependent African Americans
Source: Sci Rep. 2019 Dec 2;9:18070. doi: 10.1038/s41598-019-53560-0 (PMC6889277; doi:10.1038/s41598-019-53560-0)
Supplement: Supplementary file 1 — Supplementary Information [file 41598_2019_53560_MOESM1_ESM.docx]

**Genomewide Gene-by-Sex Interaction Scans Identify *ADGRV1* for Sex Differences in Opioid Dependent African Americans**

Bao-Zhu Yang, Hang Zhou*, Zhongshan Cheng*, Henry R. Kranzler and Joel Gelernter

* Equal contribution

**Supplementary Information**

1. METHODS
   - - The WebGestalt parameters Page 1
2. TABLES
   - - Table S1 Page 2
     - Table S2 Page 3
     - Table S3 Page 3-6
     - Table S4 Page 7
3. FIGURES
   - - Figure S1 Page 8
     - Figure S2 Page 9
     - Figure S3 Page 10
     - Figure S4 Page 11-12

**METHODS**

**The query setting parameters used for the WEB-based GEne SeT AnaLysis Toolkit (WebGestalt)**

We used the following parameters to assess the disease enrichment:

User data: textAreaUpload.txt

Organism: Hsapiens

Id Type: Entrezgene

Ref Set: Entrezgene

Significance Level: Top10

Statistics Test: Hypergeometric

MTC: BH

Minimum: 2

**TABLES**

**Table S1. The nine variants of genome-wide significance in *ADGRV1* for the sex differences in risk of opioid dependence in the African American sample.**

| **SNP** | **SNP_rsID** | **Sex** | **Three Genotypes** | | | | | | | | | **Trend Test**  ***p*-value** |
| --- | --- | --- | --- | --- | --- | --- | --- | --- | --- | --- | --- | --- |
|  |  |  | **AA** | | | **AB** | | | **BB** | | |  |
|  |  |  | **Case** | **Control** | **Prop*.** | **Case** | **Control** | **Prop.** | **Case** | **Control** | **Prop.** |  |
| **1** | **rs2030272** | **Male** | 377 | 1189 | 0.241 | 318 | 660 | 0.325 | 82 | 81 | 0.503 | 5.38E-14 |
|  |  | **Female** | 263 | 1116 | 0.191 | 137 | 595 | 0.187 | 19 | 107 | 0.151 | 0.4 |
| **2** | **rs2366929** | **Male** | 104 | 112 | 0.481 | 340 | 733 | 0.317 | 333 | 1085 | 0.235 | 1.06E-14 |
|  |  | **Female** | 25 | 142 | 0.150 | 152 | 681 | 0.182 | 242 | 995 | 0.196 | 0.15 |
| **3** | **rs2222243** | **Male** | 377 | 1188 | 0.241 | 318 | 661 | 0.325 | 82 | 81 | 0.503 | 6.28E-14 |
|  |  | **Female** | 263 | 1115 | 0.191 | 137 | 596 | 0.187 | 19 | 107 | 0.151 | 0.39 |
| **4** | **rs2443067** | **Male** | 377 | 1188 | 0.241 | 318 | 661 | 0.325 | 82 | 81 | 0.503 | 6.28E-14 |
|  |  | **Female** | 263 | 1115 | 0.191 | 137 | 596 | 0.187 | 19 | 107 | 0.151 | 0.39 |
| **5** | **rs2443066** | **Male** | 377 | 1189 | 0.241 | 318 | 660 | 0.325 | 82 | 81 | 0.503 | 5.38E-14 |
|  |  | **Female** | 263 | 1115 | 0.191 | 137 | 595 | 0.187 | 19 | 108 | 0.150 | 0.38 |
| **6** | **rs2460186** | **Male** | 376 | 1188 | 0.240 | 319 | 660 | 0.326 | 82 | 82 | 0.500 | 5.31E-14 |
|  |  | **Female** | 263 | 1113 | 0.191 | 137 | 597 | 0.187 | 19 | 108 | 0.150 | 0.36 |
| **7** | **rs2460187** | **Male** | 377 | 1190 | 0.241 | 318 | 659 | 0.325 | 82 | 81 | 0.503 | 5.31E-14 |
|  |  | **Female** | 263 | 1115 | 0.191 | 137 | 595 | 0.187 | 19 | 108 | 0.150 | 0.36 |
| **8** | **rs2443065** | **Male** | 372 | 1181 | 0.240 | 324 | 665 | 0.328 | 81 | 84 | 0.491 | 7.20E-14 |
|  |  | **Female** | 263 | 1106 | 0.192 | 135 | 599 | 0.184 | 21 | 113 | 0.157 | 0.34 |
| **9** | **rs2443064** | **Male** | 372 | 1181 | 0.240 | 324 | 665 | 0.328 | 81 | 84 | 0.491 | 7.20E-14 |
|  |  | **Female** | 263 | 1106 | 0.192 | 135 | 599 | 0.184 | 21 | 113 | 0.157 | 0.34 |

Note: *Prop., proportion, which was derived as number of cases divided by total number of cases and controls.

**Table S2. Sex differences in *ADGRV1* expression in non-ischemic human heart failure.**

| **Probe** | **Symbol ^a^** | **Average Expression ^b^** | | **Fold Change** | **Adjusted *p*-value** |
| --- | --- | --- | --- | --- | --- |
|  |  | **Females** | **Males** |  |  |
| 215396_at | *ADGRV1* | 20.6 | 18.4 | -1.25 | 7.85E-03 |

1. *ADGRV1*, *Adhesion G Protein-Coupled Receptor V1*.
2. Average expression levels are Robust Multi-array Average (RMA) normalized. The RMA procedure was primarily designed for analyzing gene expression data from Affymetrix arrays.

This table was adapted from Fermin et al. 2008. Fold changes are absolute fold changes, and adjusted p-values are FDR adjusted using the Benjamini-Hochberg procedure. The adjusted *p*-value significance level was set at *p* < 0.05.

In the original manuscript, the gene symbol they used are *GPR98 (G-Protein Coupled Receptor 98), aka. ADGRV1*, which is what we use to report our study.

Fermin DR, Barac A, Lee S, Polster SP, Hannenhalli S, Bergemann TL, Grindle S, Dyke DB, Pagani F, Miller LW, Tan S, Dos Remedios C, Cappola TP, Margulies KB, Hall JL. Sex and age dimorphism of myocardial gene expression in non-ischemic human heart failure. *Circ Cardiovasc Genet*. 2008 Dec; 1(2):117-25.

**Table S3. The top 100 co-expressed genes with *ADGRV1* identified by COXPRESdb*.**

| **Gene Symbol** | **Gene Function** | **Entrez Gene ID** |
| --- | --- | --- |
| *ADGRV1* | Adhesion G protein-coupled Receptor V1 | 84059 |
| *LOC283585* | Uncharacterized LOC283585 | 283585 |
| *OR5K1* | Olfactory receptor, family 5, subfamily K, member 1 | 26339 |
| *AREG* | Amphiregulin | 374 |
| *HOXD1* | Homeobox D1 | 3231 |
| *ABCC12* | ATP-binding cassette, sub-family C (CFTR/MRP), member 12 | 94160 |
| *OR5P2* | Olfactory receptor, family 5, subfamily P, member 2 | 120065 |
| *LOC645485* | Uncharacterized LOC645485 | 645485 |
| *TMEM144* | Transmembrane protein 144 | 55314 |
| *LACE1* | Lactation elevated 1 | 246269 |
| *LINC01538* | Long intergenic non-protein coding RNA 1538 | 400654 |
| *LRRC63* | Leucine-rich repeat containing 63 | 220416 |
| *ZSCAN23* | Zinc finger and SCAN domain containing 23 | 222696 |
| *ALG5* | ALG5, dolichyl-phosphate beta-glucosyltransferase | 29880 |
| *CFTR* | Cystic fibrosis transmembrane conductance regulator (ATP-binding cassette sub-family C, member 7) | 1080 |
| *LINC01312* | Long intergenic non-protein coding RNA 1312 | 154089 |
| *LOC145837* | Uncharacterized LOC145837 | 145837 |
| *ZNF29P* | Zinc finger protein 29, pseudogene | 7577 |
| *IQCF1* | IQ motif containing F1 | 132141 |
| *LOC100129620* | Uncharacterized LOC100129620 | 100129620 |
| *ZNF583* | Zinc finger protein 583 | 147949 |
| *MAP10* | Microtubule-associated protein 10 | 54627 |
| *CYLC1* | Cylicin, basic protein of sperm head cytoskeleton 1 | 1538 |
| *TAAR2* | Trace amine-associated receptor 2 | 9287 |
| *GRIK2* | Glutamate receptor, ionotropic, kainate 2 | 2898 |
| *TXNRD1* | Thioredoxin reductase 1 | 7296 |
| *DAOA-AS1* | DAOA antisense RNA 1 | 282706 |
| *MGAT4D* | MGAT4 family, member D | 152586 |
| *SYT16* | Synaptotagmin XVI | 83851 |
| *LINC00298* | Long intergenic non-protein coding RNA 298 | 339788 |
| *CCDC171* | Coiled-coil domain containing 171 | 203238 |
| *SPO11* | SPO11 meiotic protein covalently bound to DSB | 23626 |
| *TPH2* | Tryptophan hydroxylase 2 | 121278 |
| *ST7-OT4* | ST7 overlapping transcript 4 | 338069 |
| *RBM46* | RNA binding motif protein 46 | 166863 |
| *TRIQK* | Triple QxxK/R motif containing | 286144 |
| *OR51B5* | Olfactory receptor, family 51, subfamily B, member 5 | 282763 |
| *DNAJC9-AS1* | DNAJC9 antisense RNA 1 | 414245 |
| *FAM9B* | Family with sequence similarity 9, member B | 171483 |
| *AK8* | Adenylate kinase 8 | 158067 |
| *FLJ37201* | Tigger transposable element derived 2 pseudogene | 283011 |
| *LOC285500* | Uncharacterized LOC285500 | 285500 |
| *CRABP1* | Cellular retinoic acid binding protein 1 | 1381 |
| *LINC00610* | Long intergenic non-protein coding RNA 610 | 399879 |
| *LOC284950* | Uncharacterized LOC284950 | 284950 |
| *ZNF620* | Zinc finger protein 620 | 253639 |
| *TMEM26* | Transmembrane protein 26 | 219623 |
| *SYCP2* | Synaptonemal complex protein 2 | 10388 |
| *NAV3* | Neuron navigator 3 | 89795 |
| *RNF32* | Ring finger protein 32 | 140545 |
| *GCM2* | Glial cells missing homolog 2 (Drosophila) | 9247 |
| *VN1R2* | Vomeronasal 1 receptor 2 | 317701 |
| *PAQR9* | Progestin and adipoQ receptor family member IX | 344838 |
| *TAAR9* | Trace amine associated receptor 9 (gene/pseudogene) | 134860 |
| *LOC100130268* | Uncharacterized LOC100130268 | 100130268 |
| *FOXA1* | Forkhead box A1 | 3169 |
| *PRO2012* | Uncharacterized protein PRO2012 | 55478 |
| *LOC100131508* | PRO2122 | 100131508 |
| *CTNND2* | Catenin (cadherin-associated protein), delta 2 | 1501 |
| *LOC400748* | Uncharacterized LOC400748 | 400748 |
| *OR12D3* | Olfactory receptor, family 12, subfamily D, member 3 | 81797 |
| *FHL5* | Four and a half LIM domains 5 | 9457 |
| *ASB4* | Ankyrin repeat and SOCS box containing 4 | 51666 |
| *BCAS1* | Breast carcinoma amplified sequence 1 | 8537 |
| *EFHC2* | EF-hand domain (C-terminal) containing 2 | 80258 |
| *CAPZB* | Capping protein (actin filament) muscle Z-line, beta | 832 |
| *LOC283038* | Uncharacterized LOC283038 | 283038 |
| *ATP13A5* | ATPase type 13A5 | 344905 |
| *TAS2R8* | Taste receptor, type 2, member 8 | 50836 |
| *MAGEB4* | Melanoma antigen family B, 4 | 4115 |
| *PRO2958* | Uncharacterized LOC100128329 | 100128329 |
| *RAPGEF4-AS1* | RAPGEF4 antisense RNA 1 | 91149 |
| *ZNF287* | Zinc finger protein 287 | 57336 |
| *LOC283856* | Uncharacterized LOC283856 | 283856 |
| *SLC25A3P1* | Solute carrier family 25 (mitochondrial carrier; phosphate carrier), member 3 pseudogene 1 | 163742 |
| *TMEM92* | Transmembrane protein 92 | 162461 |
| *OR1A1* | Olfactory receptor, family 1, subfamily A, member 1 | 8383 |
| *CASC15* | Cancer susceptibility candidate 15 (non-protein coding) | 401237 |
| *RMST* | Rhabdomyosarcoma 2 associated transcript (non-protein coding) | 196475 |
| *PVT1* | Pvt1 oncogene (non-protein coding) | 5820 |
| *RSU1P2* | Ras suppressor protein 1 pseudogene 2 | 100133308 |
| *BTG4* | B-cell translocation gene 4 | 54766 |
| *DKFZp547J222* | Uncharacterized LOC84237 | 84237 |
| *ZNF157* | Zinc finger protein 157 | 7712 |
| *RNF133* | Ring finger protein 133 | 168433 |
| *ZNF560* | Zinc finger protein 560 | 147741 |
| *CCDC172* | Coiled-coil domain containing 172 | 374355 |
| *ZNF547* | Zinc finger protein 547 | 284306 |
| *AKNAD1* | AKNA domain containing 1 | 254268 |
| *TLL1* | Tolloid-like 1 | 7092 |
| *CTAGE1* | Cutaneous T-cell lymphoma-associated antigen 1 | 64693 |
| *HSD3B2* | Hydroxy-delta-5-steroid dehydrogenase, 3 beta- and steroid delta-isomerase 2 | 3284 |
| *MCF2L2* | MCF.2 cell line derived transforming sequence-like 2 | 23101 |
| *EXD1* | Exonuclease 3'-5' domain containing 1 | 161829 |
| *ADAM5* | ADAM metallopeptidase domain 5 (pseudogene) | 255926 |
| *OR5H1* | Olfactory receptor, family 5, subfamily H, member 1 | 26341 |
| *RPS6KA6* | Ribosomal protein S6 kinase, 90kDa, polypeptide 6 | 27330 |
| *FAM26D* | Family with sequence similarity 26, member D | 221301 |
| *GUSBP4* | Glucuronidase, beta pseudogene 4 | 375513 |
| *LOC339468* | Uncharacterized LOC339468 | 339468 |

* Okamura Y, Aoki Y, Obayashi T, Tadaka S, Ito S, Narise T, et al. (2015): COXPRESdb in 2015: coexpression database for animal species by DNA-microarray and RNAseq-based expression data with multiple quality assessment systems. *Nucleic Acids Res*. 43: D82-D86.

**Table S4. Disease enrichment analysis using WebGestalt 2017* among the top 100 co-expressed genes with *ADGRV1*.**

| **Disease** | **Reference genes (n)** | **Observed genes (n)** | **Expected genes (n)** | **Ratio of enrichment** | ***p*-value**** | **Adj *p****** | **Enriched genes** |
| --- | --- | --- | --- | --- | --- | --- | --- |
| Fatigue syndrome, chronic | 28 | 2 | 0.06 | 33.85 | 0.0016 | 0.0462 | *TPH2*  *GRIK2* |
| X-linked mental retardation | 46 | 2 | 0.1 | 20.61 | 0.0043 | 0.0498 | *ZNF157 GRIK2* |
| Sezary syndrome | 52 | 2 | 0.11 | 18.23 | 0.0055 | 0.0498 | *CTAGE1 NAV3* |
| Cutaneous T-cell lymphoma | 55 | 2 | 0.12 | 17.23 | 0.0061 | 0.0498 | *CTAGE1 NAV3* |
| Generalized epilepsy | 63 | 2 | 0.13 | 15.05 | 0.0079 | 0.0498 | *EFHC2 ADGRV1* |
| Pseudoxanthoma elasticum | 65 | 2 | 0.14 | 14.58 | 0.0084 | 0.0498 | *ABCC12 ADGRV1* |
| Oligospermia | 98 | 3 | 0.21 | 14.51 | 0.0012 | 0.0462 | *SYCP2*  *CFTR*  *SPO11* |
| Personality disorders | 66 | 2 | 0.14 | 14.36 | 0.0087 | 0.0498 | *TPH2*  *TAAR9* |
| Panic disorder | 121 | 3 | 0.26 | 11.75 | 0.0022 | 0.0462 | *DAOA-AS1 TPH2*  *EFHC2* |
| Infertility, Male | 188 | 3 | 0.4 | 7.56 | 0.0075 | 0.0498 | *CFTR*  *SPO11*  *FHL5* |

* Wang J, Vasaikar S, Shi Z, Greer M, Zhang B (2017). WebGestalt 2017: a more comprehensive, powerful, flexible and interactive gene set enrichment analysis toolkit. *Nucleic Acids Res* **45**(W1): W130-w137.

** Hypergeometric test *p*-value.

***Adj *p***, adjusted *p*-value for the multiple testing in the hypergeometric test.

These 13 genes are: *ABCC12*, *CFTR*, *CTAGE1*, *DAOA-AS1*, *EFHC2*, *FHL5*, *GRIK2*, *NAV3*, *SPO11*, *SYCP2*, *TAAR9*, *TPH2*, *ZNF157*.

**FIGURES**

Supplementary figures.

Figure S1.


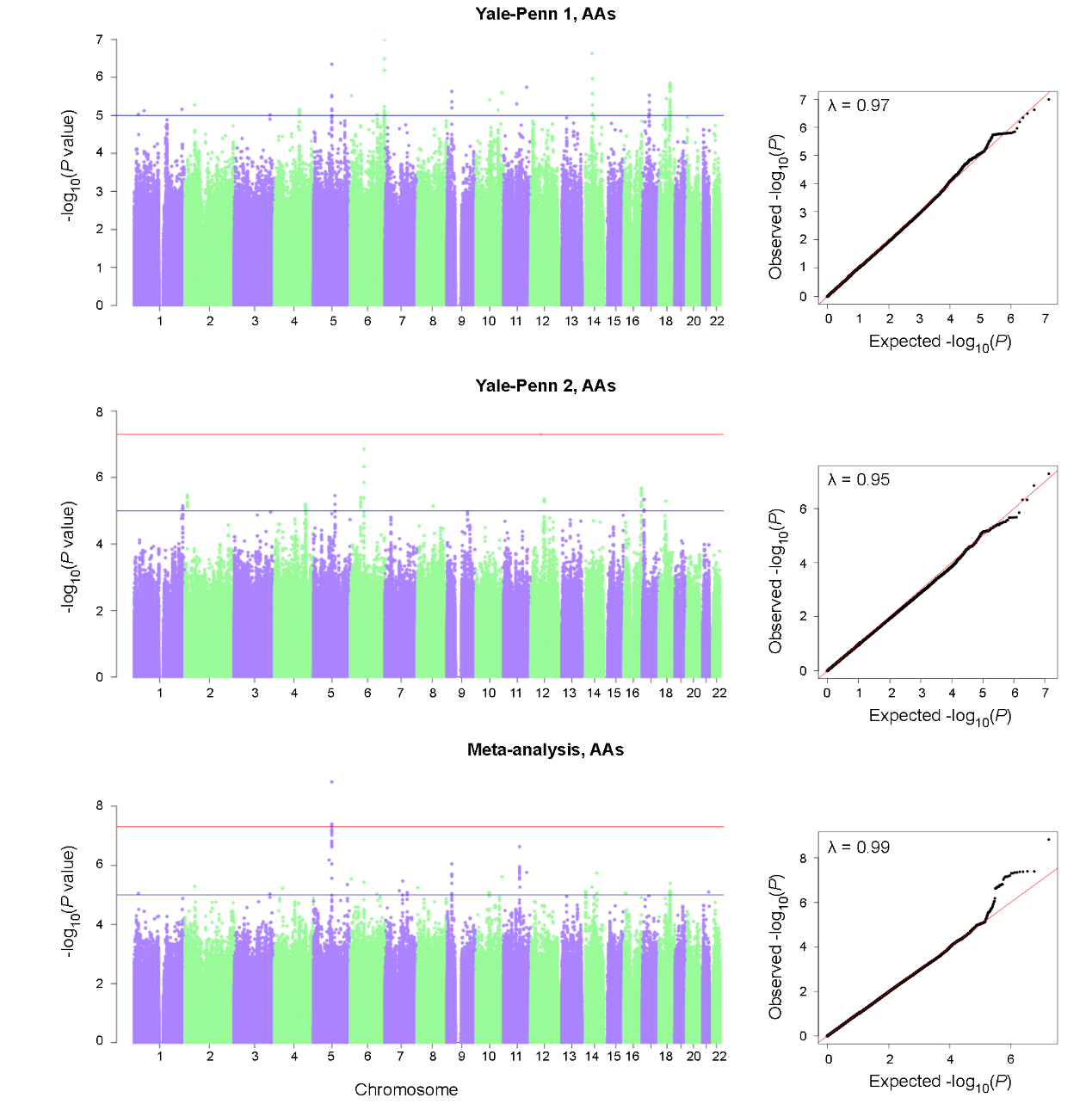


Figure S1. Genomewide gene-by-sex interaction scans for the African American (AA) samples, Yale-Penn 1 and Yale-Penn 2, and the meta-analysis of these two samples.

Figure S2.


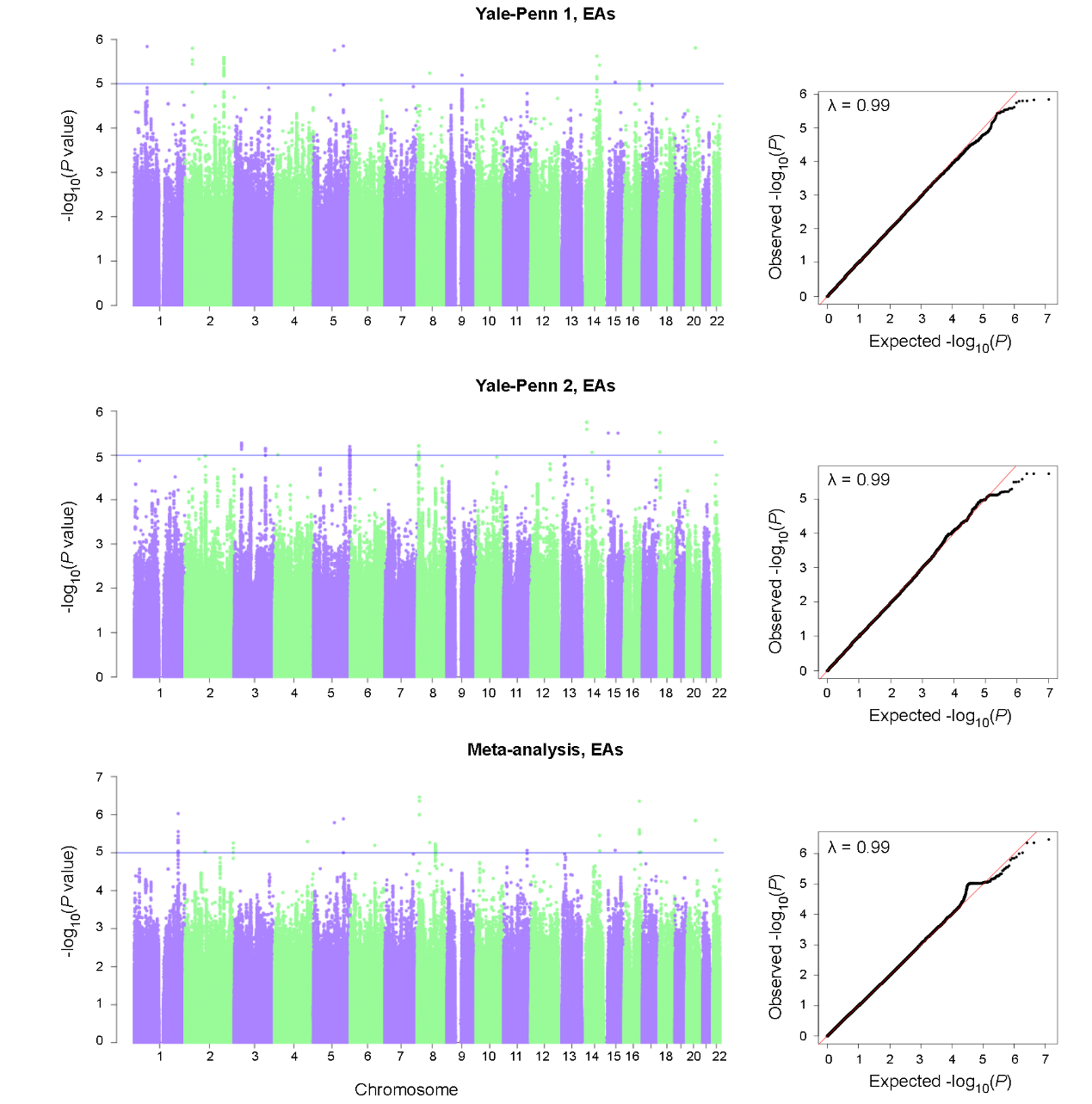


Figure S2. Genomewide gene-by-sex interaction scans for the European American (EA) samples, Yale-Penn 1 and Yale-Penn 2, and the meta-analysis of these two samples.

Figure S3.


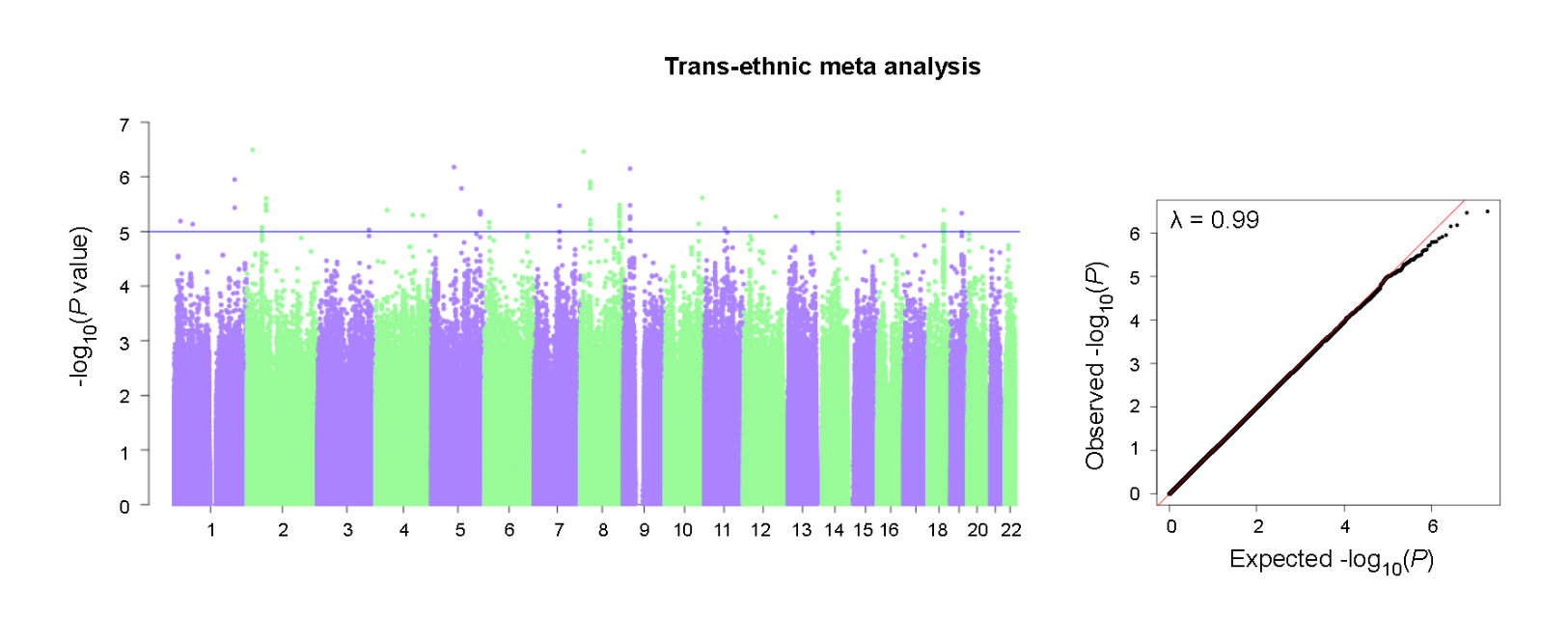


Figure S3. Genomewide gene-by-sex interaction scans for the trans-ethnic meta-analysis of the African- and European-American samples.

Figure S4.

| (A) | (B) |
| --- | --- |
| 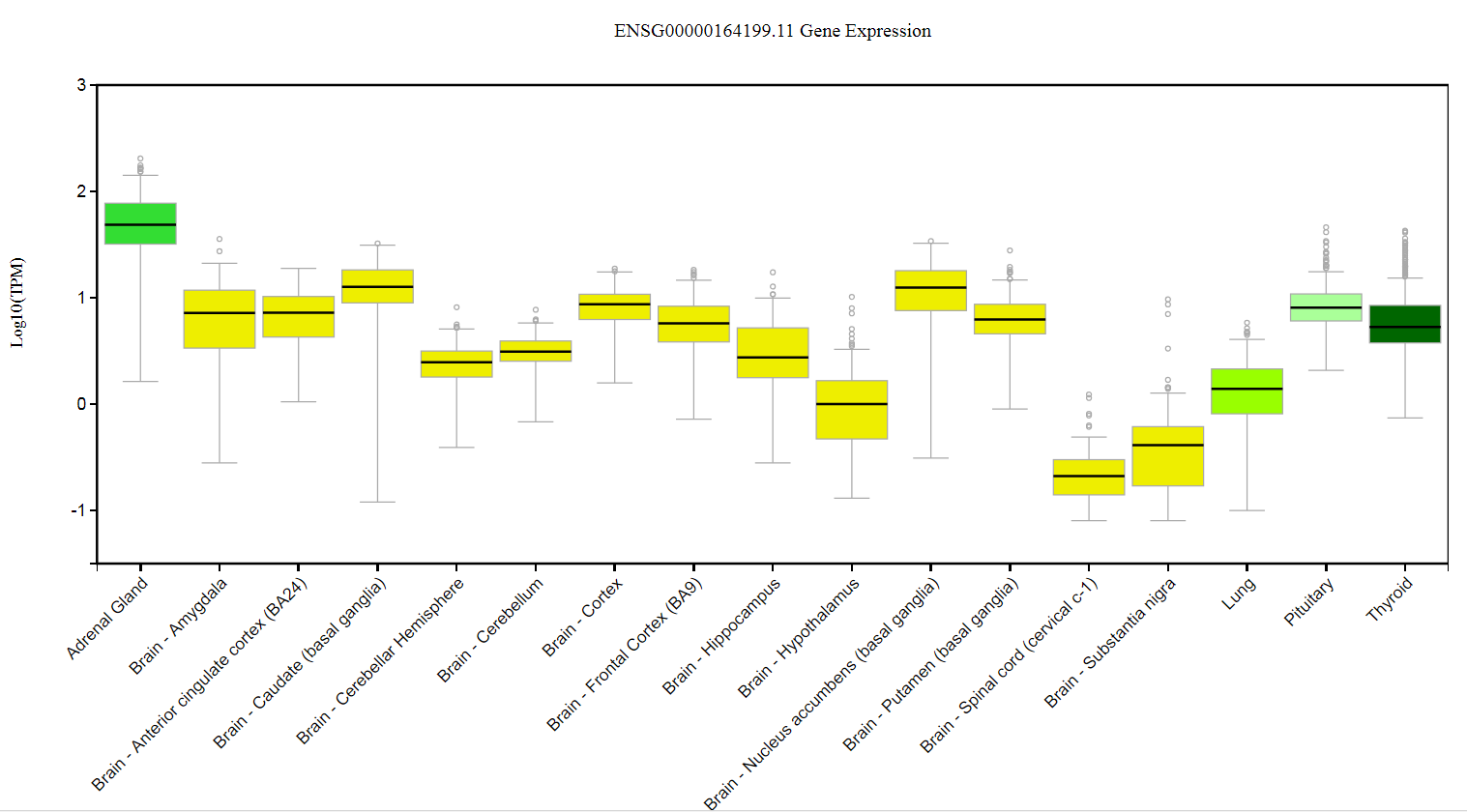 | **Left bar: RNA expression (TPM)**  **Right bar: Protein expression (score)**  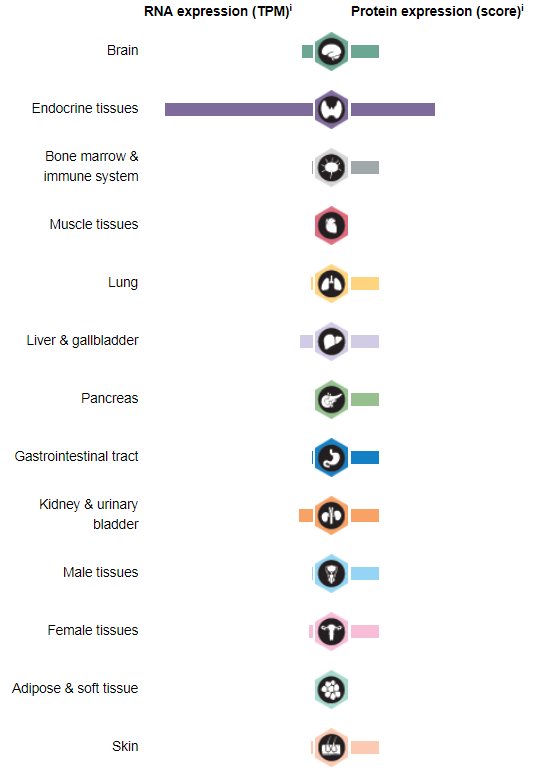 |

Figure S4. (A) *ADGRV1* gene expression: The top five tissues of *ADGRV1* expression including adrenal gland, brain, lung, pituitary, and thyroid (in which all brain regions are displayed) were selected from the Genotype-Tissue Expression (GTEx) Analysis V7 (dbGaP Accession phs000424.v7.p2). Gene expression (ENSG00000164199.11) is in transcript per million (TPM). (B). *ADGRV1* gene (left bar) and protein (right bar) expression in Human Protein Atlas (Thul PJ, et. al. 2017) (<https://www.proteinatlas.org/ENSG00000164199-ADGRV1/tissue>).

Thul PJ, Åkesson L, Wiking M, Mahdessian D, Geladaki A, Ait Blal H, Alm T, Asplund A, Björk L, Breckels LM, Bäckström A, Danielsson F, Fagerberg L, Fall J, Gatto L, Gnann C, Hober S, Hjelmare M, Johansson F, Lee S, Lindskog C, Mulder J, Mulvey CM, Nilsson P, Oksvold P, Rockberg J, Schutten R, Schwenk JM, Sivertsson Å, Sjöstedt E, Skogs M, Stadler C, Sullivan DP, Tegel H, Winsnes C, Zhang C, Zwahlen M, Mardinoglu A, Pontén F, von Feilitzen K, Lilley KS, Uhlén M, Lundberg E. A subcellular map of the human proteome. Science. 2017 May 26; 356(6340). pii: eaal3321. doi: 10.1126/science.aal3321. Epub 2017 May 11. PubMed PMID: 28495876.
